# A bibliometric analysis of tuberculosis research, 2007–2016

**DOI:** 10.1371/journal.pone.0199706

**Published:** 2018-06-25

**Authors:** Vaidehi Nafade, Madlen Nash, Sophie Huddart, Tripti Pande, Nebiat Gebreselassie, Christian Lienhardt, Madhukar Pai

**Affiliations:** 1 Department of Epidemiology, Biostatistics and Occupational Health, McGill University, Montreal, Canada; 2 McGill International TB Centre, McGill University, Montreal, Canada; 3 Global TB Programme, World Health Organization, Geneva, Switzerland; Indiana University, UNITED STATES

## Abstract

**Background:**

Tuberculosis (TB) research is a key component of the End TB Strategy. To track research output, we conducted a bibliometric analysis of TB research from the past decade.

**Methods:**

The Web of Science database was searched for publications from January 2007 to December 2016 with “tuberculosis” in the title. References were analysed using the R *bibliometrix* package. A year-stratified 5% random subset was drawn to extract funding sources and identify research areas.

**Findings:**

The annual growth rate of publications was 7.3%, and was highest (13.1%) among Brazil, Russia, India, China and South Africa (BRICS). The USA was the most productive country, with 18.4% of references, followed by India (9.7%), China (7.3%), England (6.5%), and South Africa (3.9%). In the subset analysis, the most common research area was ‘fundamental research’ (33.8%). Frequently acknowledged funders were US and EU-based, with China and India emerging as top funders. Collaborations appeared more frequently between high-income countries and low/medium income countries (LMICs), with fewer collaborations among LMICs.

**Conclusion:**

The past decade has seen a continued increase in TB publications. While USA continues to dominate research output and funding, BRICS countries have emerged as major research producers and funders. Collaborations among BRICS would enhance future TB research productivity.

## Introduction

Tuberculosis (TB) is the leading infectious disease killer, and the ninth cause of death worldwide [1]. TB mortality has been falling by about 3% per year and incidence by 2%, but the majority of countries are not on track to meet the UN 2030 Sustainable Development Goal of ending the TB epidemic by 2030 [2]. Modelling studies indicate that drastically reducing TB incidence will require new diagnostic tests, drugs, and vaccines [3]. Accordingly, intensified research and innovation is one of the three pillars of the World Health Organization’s End TB Strategy [4], and the WHO has developed the Global Action Framework for TB Research to foster high-quality TB research for the period 2016 to 2025 at global and national levels [5].

While TB incidence and mortality are regularly estimated, it is more difficult to track the evolution of knowledge generation in the field. Bibliometric analysis is a tool that has been used widely to assess research productivity and growth in the health sciences; bibliometric analyses have been published in a wide variety of research fields, such as cancer [6], respiratory medicine [7], and public health [8,9]. By conducting a statistical analysis of citations, it is possible to examine trends in research output, countries of publication, and international collaborations. A bibliometric analysis of global TB research was last published in 2008, covering research from 1997 to 2006 [10]. Since then, bibliometric analyses of drug-resistant TB [11], tubercular pleurisy [12], and spinal TB [13] have been published. Analyses of highly cited TB papers have also been published [14,15]. However, no bibliometric analysis of all TB research from 2007 onwards has been published.

Our study builds on the previous analysis of research from 1997 to 2006, and describes trends in TB research from the past decade, 2007 to 2016, through an assessment of the medical literature.

Our study aims to build on previous analyses and describe trends in TB research from the past decade, 2007 to 2016, through an assessment of the medical literature. Additionally, a random subset of articles was analysed to determine the most common areas of research and the most common sources of funding in TB research. Here we have focused on the bibliometric measures that illustrate the trajectory of TB research including which countries are producing TB research papers, the areas of research within the TB field and the major funders of this research.

## Methods

### Bibliometric analysis

We searched Web of Science on 19 July 2017 and searched for all references with ‘tuberculosis’ in the title. References published and indexed from 1 January 2007 to 31 December 2016 inclusive were included. The following information was obtained for each reference: reference type, title, journal, date of publication, author names and affiliations, and abstract.

A bibliometric analysis was performed on the full search results using the *bibliometrix* package in R [16]. This package uses the meta-data in the Web of Science citations to calculate and rank country production, journal sources, and country collaborations. Country production was defined using the first author’s country. As a sensitivity analysis, a fractional authorship attribution for the paper’s country was applied where an equal fraction of the paper was counted toward each authors' country of affiliation. This authorship assignment scheme made no meaningful difference to the results and is not shown here. Using World Bank country classification for the 2018 fiscal year, countries were divided into low, middle and high-income strata (upper- and lower-middle income countries were classified as middle income). The author affiliation strings in the Web of Science citations were searched for the presence of countries named in each classification strata. The 5-year impact factor for the top journals was obtained from Web of Science.

### Subset analysis of abstracts/full-text

Given the large numbers of references over a 10-year period, we decided to select a random subset of references to review abstracts and full-text articles. A year-stratified 5% random sample was drawn using a random number generator. This subset was screened for papers that were related to human *Mycobacterium tuberculosis* and were original research articles (i.e., not meeting abstracts, book chapters, narrative reviews, news items, editorial material and case reports/series). Systematic reviews/meta-analyses were included as original research. Data were extracted from these original research articles to determine year of publication, journal, study design, country of first author, and field-site country. The papers were then classified into one of the six research areas identified in the International Roadmap for TB Research [17], or into “other” if none of the six were appropriate. The six research areas are: fundamental research (or basic science), epidemiology, diagnostics, treatment, vaccines, and operational and public health. All screening and classification was performed independently by two authors (VN and MN), and any subsequent disagreements were resolved by consensus.

To identify sources of funding, the full text article was retrieved for the original research references and the names of all listed funding sources were extracted. Most frequently listed funders were identified overall and by research area. In the event that authors did not acknowledge funding sources in the article, or stated that no funding was received, the paper was excluded from the funding analysis.

### Ethics

As this was an analysis of available published research, no ethics approval was required. No authors were contacted for further information regarding their publications.

## Results

**Fig 1:**
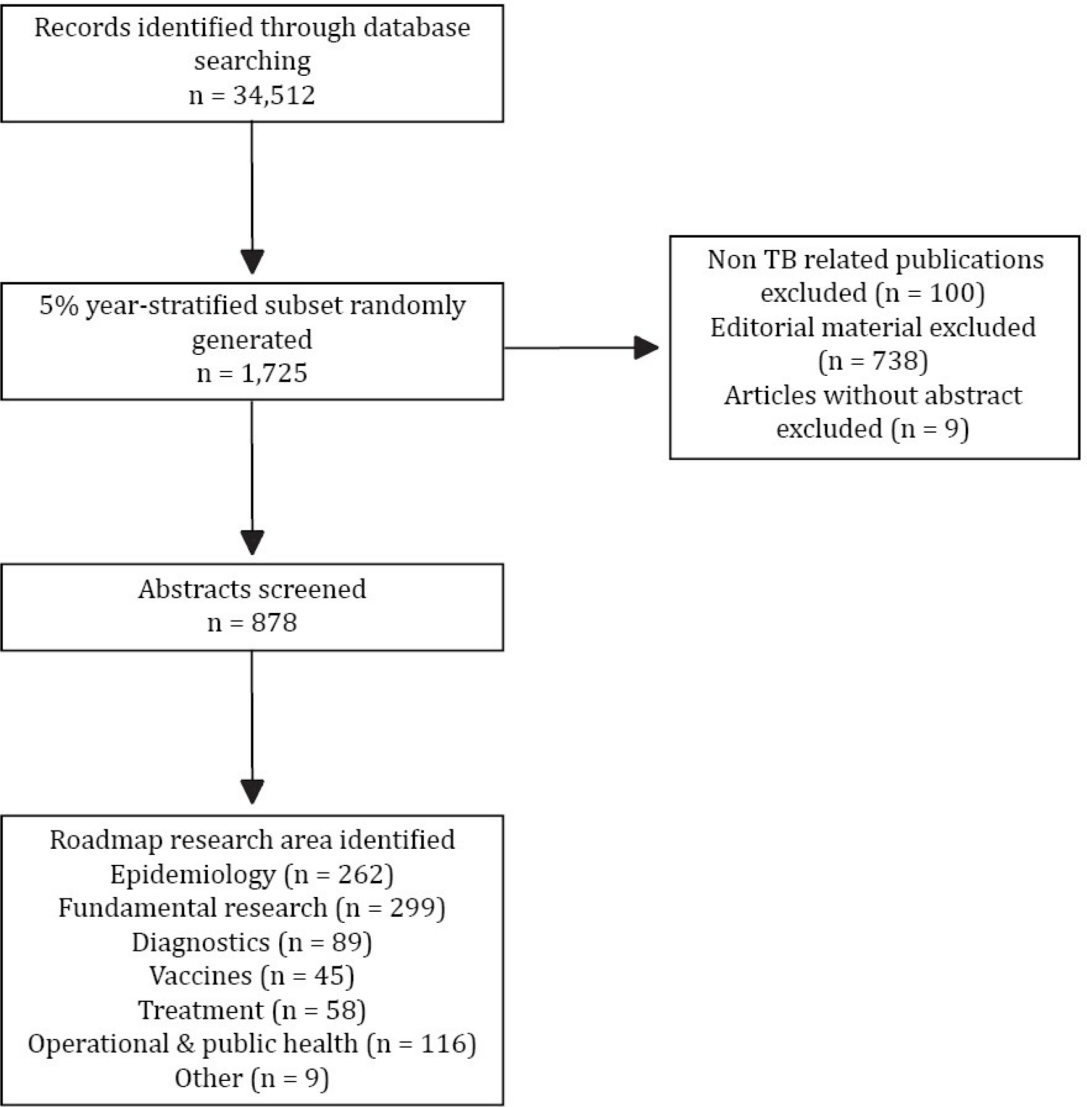
Flow chart of the bibliometric and subset analysis.

The initial search returned 34,512 references (Fig 1). Overall, both publications and inter-country collaboration have increased, with BRICS countries experiencing the greatest increase. The average year-on-year increase in publications was 7.3%. This growth rate is higher than that found in a previous bibiliometric analysis [10] of the decade 1997 to 2006, but methodological variations might confound this observation. Publications were primarily from specialty journals focused on tuberculosis, respiratory disease, or infectious disease/microbiology (Table 1); of the top ten, only *PLoS One* (ranked second) was a general journal. *The International Journal of Tuberculosis and Lung Disease* was the journal with the most published citations.

**Table 1:** Top ten journals most commonly published in over the period 2007–2016 and 5-year impact factor.

Rank	Journal	Number of articles	2016 5-year impact factor (IF)
1	International Journal of Tuberculosis and Lung Disease	2021	2.38
2	PLoS One	1726	3.39
3	American Journal of Respiratory and Critical Care Medicine	946	13.08
4	Tuberculosis	773	2.93
5	European Respiratory Journal	716	8.49
6	International Journal of Infectious Diseases	514	2.59
7	Journal of Clinical Microbiology	504	3.85
8	BMC Infectious Diseases	469	2.96
9	Respirology	404	3.18
10	Antimicrobial Agents and Chemotherapy	398	4.33

### Country of publication and growth rate

**Fig 2:**
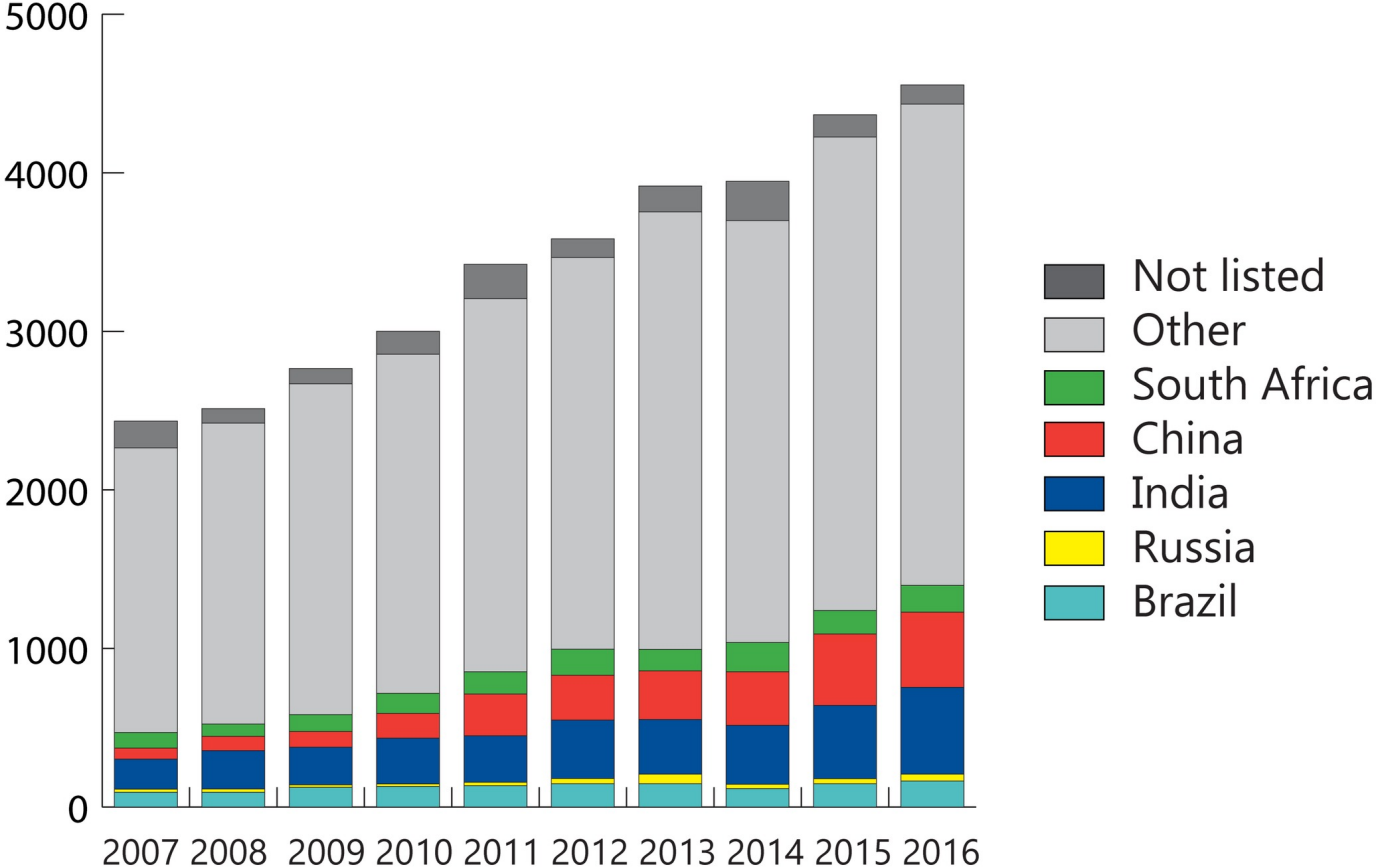
TB publications per year, 2007–2016, stratified by BRICS and non-BRICS countries.

In total, 145 unique countries were represented across the full decade, with the top ten publishing countries listed in Table 2. The United States, despite having a very low incidence of TB, produced the most publications of any one country, with 18.4% of all references. Among the top five publishing countries, three were high-burden BRICS member countries: India, China, and South Africa. Of all publications in the last decade, 25.5% had first authors from BRICS countries, the yearly proportion having increased from 19.3% in 2007 to 30.7% in 2016 (Fig 2). The average year-on-year increase in publications was 13.1% for BRICS countries, nearly double that of the increase among all countries. Out of all the BRICS countries, India, the country with the highest TB burden, had the largest proportion of publications (9.7% of total references).

**Table 2:** Top ten publishing countries in TB research, 2007–2016.

Rank	Country	n	%
1	USA	6365	18.4
2	India	3342	9.7
3	China	2534	7.3
4	England	2244	6.5
5	South Africa	1348	3.9
6	Brazil	1298	3.8
7	Spain	891	2.6
8	Korea	885	2.6
9	France	827	2.4
10	Italy	776	2.2

### Collaborations

**Fig 3:**
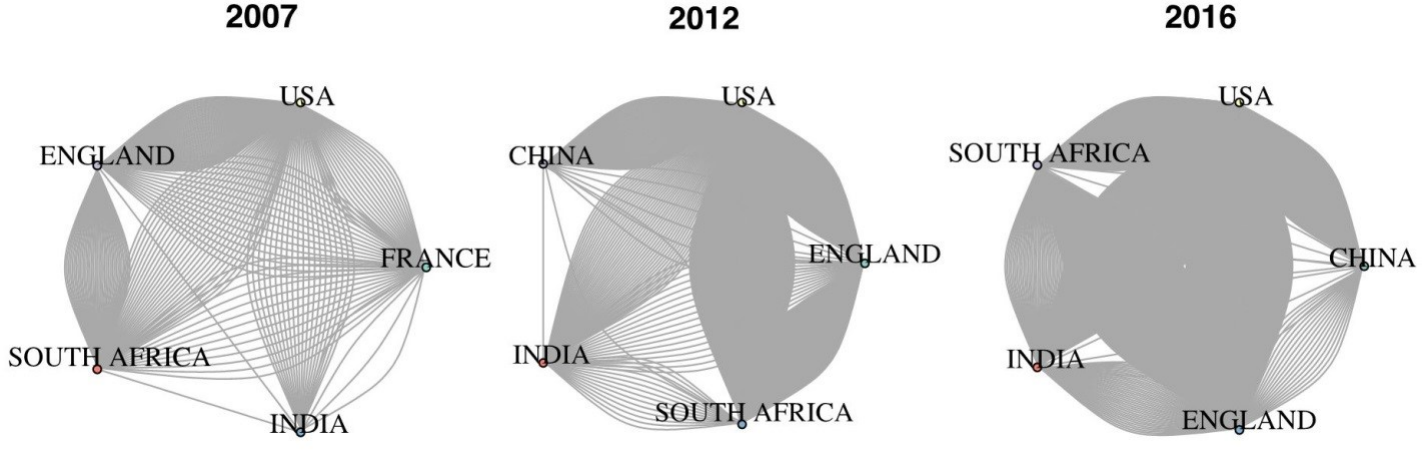
International collaborations in TB research. Each diagram includes the top five publishing countries for that year. Each line between countries represents an article with an author from each country. Dense regions of each plot indicate numerous collaborations between the two countries, demonstrating that collaborations have increased throughout the decade.

The collaboration analysis (Fig 3) demonstrates that collaborations have increased overall during the decade. Collaborations appear to be more frequent between high-income countries and LMICs; India, China, and South Africa collaborate often with the United States, but there are relatively few collaborations between the BRICS countries themselves.

### Research area

The 5% subset analysis yielded 1,725 references (Fig 1). After excluding non-original research and articles without an abstract, 878 abstracts were screened by two independent reviewers. Table 3 lists the number of articles belonging to each research area by year. ‘Fundamental research’ and ‘epidemiology’ comprised the majority of articles, with 33.8% and 29.6% of articles respectively. No significant yearly trends were identified in any research area.

**Table 3:** Proportion of original research articles in each research area by year.

Year	Epi-demiology	Fundamental research	Diagnostics	Treatment	Vaccines	Operational and PH research	Other	Number of papers
2007	32.7	26.9	7.7	7.7	7.7	13.5	3.8	52
2008	23.3	25.0	15.0	3.3	10.0	11.7	11.7	60
2009	26.4	45.8	9.7	1.4	2.8	9.7	4.2	72
2010	22.9	42.9	7.1	5.7	5.7	15.7	0.0	69
2011	26.5	38.6	12.0	2.4	6.0	13.3	1.2	83
2012	32.7	29.7	7.9	10.9	5.0	13.9	0.0	101
2013	24.5	34.9	9.4	5.7	6.6	18.9	0.0	106
2014	35.2	31.5	15.7	6.5	1.9	9.3	0.0	108
2015	29.6	35.2	7.4	10.2	3.7	13.0	0.9	103
2016	36.3	29.0	8.9	8.1	4.8	12.1	0.8	124
Overall	29.6	33.8	10.1	6.6	5.1	13.1	1.7	878

Research areas were identified from the WHO International roadmap for tuberculosis research [17].

Epidemiology: epidemiological research related to the distribution and natural history of TB

Fundamental research: basic science and laboratory research

Diagnostics: research & development of new technologies for diagnosing all forms of TB.

Treatment: research & development of new drugs and new drug regimens for treating all forms of TB.

Vaccines: research & development of new vaccines and adjuvants for preventing TB.

Operational & PH research: operational and public health research to evaluate and improve TB control programmes and design effective interventions.

Other: research that could not be categorised into one of the six research areas.

### Funding analysis

Among the subset, 30.9% of papers did not acknowledge any funding sources in the full-text article. The average number of reported funders for all papers was 1.22, and the average number of funders for papers with at least one funder listed was 1.77. The five most frequent funders by research area are listed in Table 4. Overall, major funders are US- and EU- based: National Institutes of Health (NIH), United States Agency for International Development (USAID), and the European Commission. Chinese agencies also appear on the list, particularly the National Natural Science Foundation of China, which was second overall. The Bill & Melinda Gates Foundation was third overall and among the top five for the areas of fundamental research, diagnostics, and operational and public health. Industry funders were not frequently represented overall.

**Table 4:** Top five most frequently acknowledged funders by research area in 5% subset analysis, 2007–2016.

Research area	Rank	Top funders
Epidemiology	1	National Institutes of Health, USA
	2	National Natural Science Foundation of China
	3	National Basic Research Program of China
	4	Wellcome Trust
	5	USAID
Fundamental research	1	National Institutes of Health, USA
	2	National Natural Science Foundation of China
	3	European Commission
	4	Bill & Melinda Gates Foundation
	5	Council of Scientific and Industrial Research, India
Diagnostics	1	National Institutes of Health, USA
	2	Bill & Melinda Gates Foundation
	3	Canadian Institute of Health Research
	4	European Commission
	5	USAID
Treatment	1	National Institutes of Health, USA
	2	Bristol-Myers Squibb
	3	National Natural Science Foundation of China
	4	Pfizer
	5	Project of Furong Scholar of Hunan Province
Vaccines	1	National Institutes of Health, USA
	2	European Commission
	3	Aeras
	4	National High Technology Research and Development, China
	5	National Science and Technology Major Project of China
Operational and Public Health	1	USAID
	2	National Institutes of Health, USA
	3	WHO
	4	Bill & Melinda Gates Foundation
	5	Centers for Disease Control and Prevention, USA

## Discussion

Our bibliometric analysis of TB research during the last decade shows a steady increase in TB publications. It is unsurprising that the USA had the most publications in our analysis, as it invests more in TB R&D than any other country [18]. Our findings are consistent with the previous bibliometric analysis by Ramos et al., which found that though the USA published more than any other country, its relative annual proportion of articles declined from 24.1% in 1997 to 18.4% in 2006 [10]. The trend of increased research output from BRICS countries is encouraging, as these five countries cumulatively account for 38% of the world’s notified TB cases [1].

BRICS countries–particularly India, South Africa, and China–have rapidly developing economies, and their domestic research capacity and impact on global health research appears to have increased accordingly [19]. In fact, 46% of global funding for TB R&D in 2017 is in BRICS countries, although their spending on TB R&D still does not meet set targets [1]. The identified proportion of governmental funding agencies from India and China in our funding analysis is consistent with this, and also with our finding that India and China are among the most productive countries in terms of publication number. Progress towards eliminating TB will require BRICS countries to sustain this observed increase in TB research, and ensure that research is also translated into effective policy.

The 5% sample size limits the generalisability of our results, particularly in the funding analysis where data were sparser, further decreasing the sample size and limiting interpretability of the funding results. Further, our analysis only determines the proportion of articles listing a particular funding agency as a source, and not the size of the financial contribution. The cost of one study is not equal across different types of studies. However, our results are in line with what is known about global funding for TB R&D; among TB research funding institutions, the NIH, USAID, and Bill & Melinda Gates Foundation have been top funders across the past decade [18].

Despite the increase in research output, our results suggest that collaborations among BRICS countries occur much less frequently than collaborations between a BRICS member and a high-income, low-burden nation such as the U.S. or U.K. As TB R&D in BRICS is primarily funded by domestic sources, these countries potentially have the capacity to collaborate without funding from high-income countries [1]. While north-south collaborations allow for the sharing of valuable expertise as well as resources, the trend of increased collaboration overall will ideally be followed by one of increased collaboration among LMICs.

It is heartening to note that the Moscow Declaration to End TB called for WHO in collaboration with global partners, research organizations, donors, the scientific community and countries to consider developing a Global Strategy for TB Research taking into consideration ongoing and new efforts, such as the TB Research Network stated in the BRICS Leaders Xiamen Declaration [20]. A TB Research Network by BRICS would go a long way in enhancing collaborations among these countries and harmonize research funding.

A major limitation of our study is that neither the bibliometric nor the subset analysis effectively capture the quality of publications. Our results indicated that most TB research is published in low to modest impact factor journals, but journal impact factor does not necessarily reflect the quality of an article [21]. Previous attempts to capture article quality have also focused on citations; for example, an analysis of TB research in India from 1998 to 2009 analysed citations per paper for BRICS countries and found that articles from Brazil and South Africa received more citations on average than articles from India [22]. As such, there was no effective measure of article quality in our analysis.

Another limitation is that the affiliation address of the first author does not necessarily reflect the country where the research was conducted, nor the originator of the research project. Especially in the case of collaborative papers, the principal investigator and source of funding may be in a different country than the first author. However, a sensitivity analysis using fractional authorship attribution did not significantly change our results. Further, Web of Science may capture fewer non-English publications than other databases such as PubMed [1]. As a result, articles from English-speaking countries–which are primarily high-income and low-burden–may be overrepresented. Researchers in smaller countries with a lower research capacity may be more likely to publish in non-English journals that are only indexed in regional databases. Additionally, developing countries are often underrepresented in major medical journals [23].

The most cited articles were not examined in this analysis. We did not include this as detailed analyses of the 100 most cited studies [14] and 100 most cited systematic reviews [15] in TB research have been published in the last five years.

## Conclusions

Publications related to TB have increased over the past decade, particularly among BRICS countries, though the U.S. and European countries remain a major source of publications and funding. International research collaborations have increased as well, though North-South collaborations remain more common than South-South collaborations. Our findings are consistent with previous bibliometric analyses in the field of TB research that have described overall growth in publications [10,11] and collaborations [11].

Our findings informed the recently published policy paper entitled “Global investments in Tuberculosis research and development: past, present and future,” published by WHO at the Global Ministerial Conference on Ending TB in the Sustainable Development Era, held in Moscow, Russia, on November 16–17, 2017 [24]. It is expected that this report will advance the global TB research agenda, and contribute to the goals of the End TB Strategy.
